# Medications as a Trigger of Sleep-Related Eating Disorder: A Disproportionality Analysis

**DOI:** 10.3390/jcm11133890

**Published:** 2022-07-04

**Authors:** Diane Merino, Alexandre O. Gérard, Elise K. Van Obberghen, Nouha Ben Othman, Eric Ettore, Bruno Giordana, Delphine Viard, Fanny Rocher, Alexandre Destere, Michel Benoit, Milou-Daniel Drici

**Affiliations:** 1 Department of Psychiatry, University Hospital of Nice, 06000 Nice, France; merino.d@chu-nice.fr (D.M.); ettore.e@chu-nice.fr (E.E.); giordana.b@chu-nice.fr (B.G.); benoit.m@chu-nice.fr (M.B.); 2 Department of Pharmacology and Pharmacovigilance Center of Nice, University Hospital of Nice, 06000 Nice, France; gerard.a@chu-nice.fr (A.O.G.); ben-othman.n@chu-nice.fr (N.B.O.); viard.d@chu-nice.fr (D.V.); rocher.f@chu-nice.fr (F.R.); destere.a@chu-nice.fr (A.D.); 3 Department of Nephrology-Dialysis-Transplantation, University Hospital of Nice, 06000 Nice, France; 4 Department of Neurology, University Hospital of Nice, 06000 Nice, France; vanobberghen-blanc.e@chu-nice.fr

**Keywords:** antipsychotics, amphetamines, hypnotics, sleep-related eating disorder, adverse drug reaction, clinical epidemiology

## Abstract

Sleep-related eating disorder (SRED) is a parasomnia with recurrent, involuntary, amnestic eating episodes during sleep. There is growing evidence of the association between SRED and medications. Therefore, we aimed to rank drugs showing the strongest association. VigiBase^®^ (WHO pharmacovigilance database) was queried for all reports of “Sleep-related eating disorder”. Disproportionality analysis relied on the Reporting Odds Ratio, with its 95% Confidence Interval (CI), and the Information Component. Our VigiBase^®^ query yielded 676 cases of drug-associated SRED. Reports mostly involved zolpidem (243, 35.9%), sodium oxybate (185, 27.4%), and quetiapine (97, 14.3%). Significant disproportionality was found for 35 medications, including zolpidem (387.6; 95%CI 331.2–453.7), sodium oxybate (204.2; 95%CI 172.4–241.8), suvorexant (67.3; 95%CI 38.0–119.2), quetiapine (53.3; 95%CI 43.0–66.1), and several psychostimulants and serotonin-norepinephrine reuptake inhibitors (SNRIs). Patients treated with nonbenzodiazepines or SNRIs were significantly older (mean age: 49.0 vs. 37.5; *p* < 0.001) and their SRED were more likely to be serious (62.6% vs. 51.4%; *p* = 0.014) than patients treated with sodium oxybate or psychostimulants. Psychotropic drugs are involved in almost all reports. In patients with SRED, an iatrogenic trigger should be searched for.

## 1. Introduction

Eating disorders often foster sleep disturbances [1]. Conversely, sleep disorders may interfere with eating behaviors. Both may interact to yield complex sleep behaviors (CSBs), including sleep-related eating disorder (SRED) [2].

SRED is a parasomnia defined by recurrent, involuntary, and amnestic eating episodes while asleep, with frequent consumption of unpalatable foods. With an estimated prevalence of up to 5%, SRED mostly affects young adult females, but the age range extends from adolescence to late middle age [3]. Diagnostic criteria for SRED were updated in 2014 by the American Academy of Sleep Medicine [4]. SRED is characterized by dysfunctional eating behaviors that occur after an arousal during the main sleep period, associated with at least one of the following signs: consumption of peculiar forms or combinations of food or inedible or toxic substances, sleep-related injurious or potentially injurious behaviors performed while in pursuit of food or while cooking food, and/or adverse health consequences from recurrent nocturnal eating (such as weight gain) [1]. There is a partial or complete loss of conscious awareness during the eating episode, with subsequent impaired recall. Compared with other non-rapid eye movement (NREM) parasomnias, the larger variability in patient awareness during SRED episodes sometimes represents a critical issue in the differential diagnosis with nocturnal eating syndrome (NES) [2]. However, the underlying mechanisms might differ from those of NES [5], which is characterized by recurrent episodes of eating after the evening meal or after awakening from sleep, and belongs to the “other specified feeding or eating disorder” category in the Diagnostic and Statistical Manual of Mental Disorders, Fifth Edition (DSM V) [2].

By contrast, promoters of SRED include sleepwalking, restless legs syndrome, obstructive sleep apnea, various causes of sleep fragmentation, delayed circadian rhythm of eating, daytime eating disorders, and increased central dopamine release [4,6,7,8,9].

Medication-associated SRED (e.g., zolpidem, quetiapine) has been increasingly reported in case series and case reports [8,10,11,12,13,14,15,16]. Therefore, we aimed to seek for potential safety signals regarding the association of SRED with drugs in the World Health Organization (WHO) database. The clinical profiles of affected patients were also analyzed.

## 2. Materials and Methods

### 2.1. Data Source

The Uppsala Monitoring Centre (UMC) has been mandated by the WHO to oversee drug safety since 1978. The UMC database collects data issued by the 172 national pharmacovigilance network members, and pharmaceutical laboratories. VigiBase^®^ [17] (WHO pharmacovigilance database) gathers spontaneous reports, ensuring the preservation of the anonymity of both patients and notifiers.

Sociodemographic characteristics (age, sex, area of residence, notifier’s country) and details concerning the notified effect (suspected drugs, concomitant drugs, adverse drug reaction, date of occurrence, and seriousness) are collected in the database.

### 2.2. Query

VigiBase^®^ was queried for all reports of the Preferred Term “Sleep-related eating disorder” (Medical Dictionary for Regulatory Activities, MedDRA 24.1 [18]), registered between 14 November 1967 (first reports in VigiBase^®^) and 30 December 2021. A preferred term (PT) designates a single medical concept in the most clinically precise way. The term ‘Active Ingredients’ designates all the drugs included in the database (classified by molecules’ names). Aiming to mitigate the risk of artefactual disproportionality signals, which can arise when a drug is involved in very few cases of a given adverse drug reaction, all Active Ingredients deemed suspect or interacting in at least 3 cases were included.

Quantitative variables were described in terms of medians with interquartile range (IQR) or means with their standard deviations (±SD). Qualitative variables were described in terms of effectives and proportions. Characteristics of patients were compared using Pearson’s Chi-Square test for categorical variables, or Student’s t-test for normally distributed continuous variables. *p* < 0.05 was considered to be statistically significant. We used GraphPad Prism version 8.0.2 software to determine medians with their IQRs, means with their SDs, and to perform Pearson’s Chi-Square test and Student’s *t*-test.

### 2.3. Disproportionality Analysis

Next, we sought a potential pharmacovigilance signal relying on a disproportionality approach for all drugs. We used the analytic tools provided by Vigilyze on its website, which rely on disproportionality approaches, such as calculation of the Reporting Odds Ratio (ROR) and the Information Component (IC) to allow data mining in VigiBase^®^.

As the approximate of the odds ratio (used in case-control studies) in case–non-case studies, the ROR assesses the strength of disproportionality. In VigiBase^®^, for every drug-ADR association, all the other drugs and ADRs reported in the database are used as comparators in the disproportionality analysis, to assess whether the ADR is disproportionately reported with this specific drug. A ROR equal to 1 indicates the absence of signal: the adverse drug reaction (ADR) is similarly reported with the drug of interest as with other drugs. Conversely, a ROR greater than 1 reveals a signal, cases being more frequently reported with the drug of interest than with others. The higher the ROR, the greater the association. The ROR has to be interpreted with its 95% confidence interval (95%CI). Consequently, a ROR is deemed statistically significant when the lower bound of its 95% CI is greater than 1 [19].

To yield associations, IC compares observed and expected values for the combination of a chosen drug and an ADR. It lowers the risk of false-positive signals, especially if the given ADR has a very low expected frequency in the database (artificially increasing the ROR). The positivity of the IC indicates the superiority of the number of observed reports over their expected number. IC025 is the bottom end of the 95% CI of the Information Component. For UMC, a positive IC025 is required to define a statistically significant signal [17].

In this disproportionality analysis, relevant associations were chosen using IC025. Then, Active Ingredients were ranked according to their association with SRED, via their ROR.

## 3. Results

### 3.1. Characteristics of the Reports

As of 30 December 2021, we retrieved 676 cases of sleep-related eating disorder (PT) in VigiBase^®^. The majority of these notifications (573 reports, 84.8%) originated from the United States. When mentioned, the notifier was mostly a healthcare professional (301 reports, 44.5%). Most patients were females (415, 61.4%). The age range most represented was the 45-to-64-year group, with 174 (25.7%) reports, and the median age was 46 years (IQR: 33–55).

The median time to onset was 61 days (IQR: 4–366). SRED was considered to be serious in 343 (50.7%) reports, including 23 (3.4%) life-threatening reactions. These cases were mainly linked to abuse of recreational drugs or traumatic consequences of associated CSBs. “Somnambulism” (316, 46.7%), “weight increased” (127, 18.8%), and “amnesia” (118, 17.5%) were the most frequent co-reported MedDRA clinical terms. The outcome was available in 318 cases (47.0%). Among cases with available outcomes, most patients recovered (196, 61.6%). A total of 19 (6.0%) were recovering or recovered with sequelae, and 103 (32.4%) patients did not recover at all. Detailed characteristics of the reports are provided in Table 1.

### 3.2. Active Ingredients Ranked by Absolute Number of Reports

Drugs from the “Nervous system” (N) drug class, in the Anatomical Therapeutic Chemical (ATC) classification system, were involved with almost all SRED reports (654, 96.7%).

The active ingredients most frequently reported as suspect or interacting belonged to the nonbenzodiazepine class, with 259 (38.3%) reports. Indeed, zolpidem accounted for more than one-third of the reports (243, 35.9%). Patients treated with nonbenzodiazepine drugs were mostly females (70.4%), with an average age of 49.7 years (±16.6), and the SRED was considered to be serious in most (64.0%) cases.

Sodium oxybate (sodium salt of γ-hydroxybutyric acid, GHB) was involved in more than one-quarter of the cases (185, 27.4%). Those reports involved a majority of women (68.2%), with a mean age of 39.7 years (±17.4) and a share of serious reports of 48.6%.

Atypical antipsychotics (AAPs), mostly represented by quetiapine (97, 14.3%), accounted for more than a fifth of the cases (141, 20.8%). Among cases related to AAPs, patients mostly included females (57.7%), with an average age of 41.1 years (±14.5). These SRED reports were deemed to be serious in 45.2% of cases.

Serotonin-norepinephrine reuptake inhibitors (SNRIs), duloxetine and venlafaxine, represented 18 (2.7%) and 14 (2.1%) reports, respectively. Those reports involved a significant share of women (68.3%), with a mean age of 44.8 years (±15.6) and a majority (62.3%) of serious cases.

Among psychostimulants, (dex)amfetamine was involved in 10 (1.5%) reports, followed by lisdexamfetamine (7, 1.0%), armodafinil, methylphenidate, and phentermine with 5 (0.7%) reports each, and modafinil (3, 0.4%). Reports with psychostimulants involved 65.0% of females, with an average age of 37.2 (±15.8) and 54.3% of serious cases.

Patients treated with nonbenzodiazepines or SNRIs were significantly older (mean age: 49.0 ± 15.0 vs. 37.5 ± 17.2; *p* < 0.001) and their SRED cases were more likely to be serious (62.6% vs. 51.4%; *p* = 0.014) than patients treated with sodium oxybate or psychostimulants.

Characteristics of the SRED reports according to drug classes are summarized in Table 2. A more comprehensive list of active ingredients is provided in Table S1.

### 3.3. Disproportionality Analysis

Significant disproportionality was found for 35 active ingredients with an IC025 > 0, ranked according to their ROR as shown in Table 3.

Hypnotics were associated with the strongest RORs. Zolpidem, with a ROR of 387.6 (95%CI 331.2–453.7), ranked first. Sodium oxybate, a gamma-aminobutyric acid (GABA) type B agonist was associated with a strong ROR (204.2; 95%CI 172.4–241.8). The other nonbenzodiazepines hypnotics, zopiclone and eszopiclone, yielded RORs of 43.6 (95%CI 24.6–77.2) and 15.6 (95%CI 5.8–41.6). Suvorexant, an orexin antagonist hypnotic, was associated with a ROR of 67.3 (95%CI 38.0–119.2).

Among AAPs, quetiapine was characterized by the highest ROR (53.3; 95%CI 43.0–66.1), followed by ziprasidone (16.9; 95%CI 7.6–37.7), aripiprazole (15.4; 95%CI 10.2–23.1), lurasidone (10.3; 95%CI 3.3–31.9), and olanzapine (6.6; 95%CI 3.6–12.0).

RORs for psychostimulants armodafinil (40.8; 95%CI 16.9–98.4), (dex)amfetamine (33.4; 95%CI 17.9–62.4), modafinil (24.0; 95%CI 7.7–74.8), lisdexamfetamine (20.3; 95%CI 9.6–42.8), phentermine (10.2; 95%CI 4.3–24.7), and methylphenidate (4.6; 95%CI 1.9–11.0) were also significant.

The SNRIs duloxetine and venlafaxine also had significant disproportionate reporting, with RORs of 11.3 (95%CI 7.1–18.1) and 8.8 (95%CI 5.2–15.0).

## 4. Discussion

Almost all reports of drug-related SREDs in the WHO pharmacovigilance database involve psychotropic drugs. Although we confirm its female predominance [3,9,16], patients with drug-induced SRED were older than expected [3].

Two main profiles stand out. SRED cases related to nonbenzodiazepines and SNRIs involve older patients and are deemed to be serious more frequently. Indeed, these drugs are mostly prescribed to the elderly [20,21], which may account for the increased severity of their SRED reports. Zolpidem, zopiclone, and eszopiclone have been subject to an alert from the Food and Drug Administration (FDA), which reported rare, but serious events caused by CSBs [22]. We concur with this alert, as SRED is frequently associated with sleep-walking, sleep-driving, or sleep-sex [23]. Most fatal cases reported in VigiBase^®^ were not directly imputable to SRED *per se*, but rather to concurrent CSB-related injuries. However, fatal consumption of dangerous substances during SRED episodes is not rare [24,25]. The amnesic properties of nonbenzodiazepines and SNRIs may increase the risk of such iterative dangerous behaviors [26,27].

By contrast, patients treated with sodium oxybate and psychostimulants are younger, and their SRED reports were less likely to be serious. In fact, sodium oxybate [28] and psychostimulants [29] are conducive to intentional misuse and abuse. The latter suggests a more occasional use of these drugs, therefore lowering the number of events in patients suffering from SRED.

Predictably, hypnotics represent a major share of SRED reports in our analysis. Indeed, increased GABAergic activity (by nonbenzodiazepines) may promote disinhibition of eating behavior. Benzodiazepines are less represented, in terms of number of reports and disproportionality. This may reflect the fact that nonbenzodiazepines are more frequently prescribed that benzodiazepines in insomnia [30]. Besides, nonbenzodiazepines act on selective benzodiazepine receptor sites in the GABA_A_-receptor complex [31]. This specificity, while reducing the global neurologic ADR risk, could also increase the duration of slow-wave sleep [31], therefore fostering SRED [1]. In addition, zolpidem has been found to increase the risk of other NREM parasomnias (potentially leading to various CSBs), suggesting a shared pathophysiology [3]. Zolpidem seems to exert more specific effects on thalamocortical relay neurons (due to a higher GABA α1 affinity), while eszopiclone would more likely affect interneurons of the reticular thalamic nucleus [4]. This may contribute to some clinical differences between zolpidem and zopiclone and derivatives, including SRED promotion.

The orexin antagonism induced by the hypnotic suvorexant could fragment non-rapid eye movement sleep, therefore fostering parasomnias such as SRED [6,32,33]. Sodium oxybate may also trigger SRED, by enhancing cortical dopamine release [34], or more likely by increasing slow-wave sleep duration and delta power [6]. Indeed, the labels of these drugs mention complex sleep behaviors [26,35,36].

One-fifth of the SRED cases are related to AAPs. Quetiapine, ziprasidone, aripiprazole, lurasidone, and olanzapine have been associated with SRED [37,38]. Quetiapine stands out in terms of number of cases and disproportionality. AAPs block 5-hydroxytryptamine-2A (5HT-2A) and dopamine receptors subtype 2, which enhances dopamine release [39]. 5HT-2A receptors, in the dorsal raphe nucleus, modulate periodicity and amplitude of slow-wave sleep [40]. Both mechanisms concur with a possible serotonin influence on SRED [11], while AAP-induced appetite stimulation may constitute an important confounding factor [41]. In fact, AAPs might increase the degree of hunger, while lowering the satiating efficiency [7]. Further, patients treated with AAPs may show tendencies for recurrent episodes of dietary disinhibition, which may favor proper eating disorders [8]. Indeed, the involvement of serotonin and histamine in the control of appetite is well known, and H1 receptor blockade showed the strongest association with body weight gain [9]. In addition, H1 receptor blockade may also facilitate NREM sleep [5,10], which supports a histaminergic role in the modulation and sleep and arousal. In this context, histamine dysregulations may favor the occurrence of SRED.

However, risperidone [15] and lithium [24] were not disproportionately associated with SRED in our analysis.

Our study highlights the role of several psychostimulants. Pharmacodynamics of amphetamines (amfetamine, dexamfetamine, lisdexamfetamine, phentermine) and derivatives (methylphenidate) feature dopamine reuptake inhibition [42] and direct catecholamine release [43]. Non-amphetaminic psychostimulants (armodafinil, modafinil) also modulate dopamine metabolism [44]. Both pharmacological hypotheses may be involved in the deregulation of the sleep-wake cycle, potentially triggering parasomnias such as SRED [45]. This result may be confounded by the fact that amphetaminic stimulants are used in patients with hypersomnia, which is sometimes comorbid with SRED [46]. Besides, the catecholaminergic hypothesis may underlie cases of SRED involving SNRIs [45]. Indeed, results are conflicting regarding the promoter role of noradrenaline in hypophagia and hyperphagia, highlighting its potential specific actions in influencing human eating behavior [11]. While some studies demonstrated the role of the noradrenergic system in binge-like behaviors, with a key activation of stress circuits, others suggest the existence of a noradrenergic-mediated genetic susceptibility among patients suffering from anorexia nervosa. Besides, pharmacological studies found an efficacy of SNRIs in the treatment of bulimia nervosa and binge eating disorder [11]. Regarding sleep, noradrenergic neurons in the locus coeruleus [12] belong to the ascending reticular activating system, which controls waking. During sleep, the waking noradrenergic system is inhibited via a GABAergic mediation. Conversely, sleep-active neurons are inhibited by noradrenaline, via postsynaptic α2-adrenoceptors [13]. In this context, a noradrenergic modulation might affect both eating and sleeping behaviors. By contrast, serotonin selective reuptake inhibitors are sometimes used in the treatment of SRED, which may constitute a confounding factor [47,48].

This comprehensive analysis of SRED reports from the WHO safety database is hindered by some limitations. First, clinicians are prone to ascribe a SRED diagnosis to a drug already known to cause SRED, which constitutes a reporting bias. Second, SRED belongs to parasomnias and under-reporting may be a prevalent limitation, as its diagnosis requires patients’ awareness and/or bedpartner or roommate observation. Third, coding heterogeneity reflects the lack of standardization of diagnosis among reports. Indeed, the possibility that some reports were wrongly attributed to iatrogenic triggers cannot be ruled out, especially in patients intrinsically at high risk of SRED. The disproportionality analysis was intended to partly balance some of those confounding factors. Incomplete data being inherent to postmarketing pharmacovigilance studies, available clinical data are too scarce to distinguish with certainty an aggravated preexisting SRED from a *de novo* SRED. Likewise, heterogeneity in the coding of outcomes prevents from precisely assessing the reversibility of SRED. In any case, pharmacovigilance studies are exploratory by nature, aiming to raise awareness by suggesting potential drug signals, and do not allow to conclude on a causal association between an effect and a given drug. The causal link between SRED and psychotropic drugs must be further confirmed.

## 5. Conclusions

In this study, based on a global analysis of SRED reports gathered into the WHO safety database, we suggest potential pharmacovigilance signals involving several drugs, mainly belonging to hypnotics, atypical antipsychotics, psychostimulants, and serotonin-norepinephrine reuptake inhibitors. These findings may strengthen growing evidence regarding the association of SRED with nonbenzodiazepines and atypical antipsychotics. In patients with SRED, an iatrogenic trigger should be searched for.

## Figures and Tables

**Table 1:** Characteristics of the reports of patients with sleep-related eating disorder (SRED).

Characteristics	Number (%)
**Total**	676 (100)
Female	415 (61.4)
Male	202 (29.9)
Unknown	59 (8.7)
**Age**	
2–11 years	6 (0.9)
12–17 years	10 (1.5)
18–44 years	168 (24.9)
45–64 years	174 (25.7)
65–74 years	16 (2.4)
≥75 years	17 (2.5)
Unknown	285 (42.2)
**Country**	
United States of America	573 (84.8)
Australia	26 (3.8)
Spain	21 (3.1)
Canada	12 (1.8)
United Kingdom	12 (1.8)
France	4 (0.6)
Italy	4 (0.6)
Japan	4 (0.6)
Sweden	4 (0.6)
Hungary	3 (0.4)
Turkey	3 (0.4)
Germany	2 (0.3)
Norway	2 (0.3)
Switzerland	1 (0.1)
Denmark	1 (0.1)
Greece	1 (0.1)
Netherlands	1 (0.1)
Portugal	1 (0.1)
Slovakia	1 (0.1)
**Reporter qualification**	
*Healthcare Professional*	300 (44.4)
Physician	143 (21.2)
Pharmacist	19 (2.8)
Other Health Professional	138 (20.4)
*Others*	259 (38.3)
Lawyer	5 (0.7)
Consumer/Non-Health Professional	254 (37.6)
Unknown	117 (17.3)

**Table 2:** Characteristics of the reports of patients with sleep-related eating disorder (SRED) according to drug classes.

Drug Class	Age (Mean ± SD)	Female (%)	Serious (%)
Nonbenzodiazepines	49.7 (±16.6)	70.4	64.0
Serotonin-norepinephrine reuptake inhibitors	44.8 (±15.6)	68.3	62.3
Second-generation antipsychotics	41.1 (±14.5)	57.7	45.2
Sodium oxybate	39.7 (±17.4)	68.2	48.6
Psychostimulants	37.2 (±15.8)	65.0	54.3

SD: Standard Deviation.

**Table 3:** Disproportionality analysis for reports of sleep-related eating disorder (SRED).

Active Ingredient	ROR	95% CI	Number of Cases (%)
Zolpidem	387.6	331.2–453.7	243 (35.9)
Sodium Oxybate	204.2	172.4–241.8	185 (27.4)
Suvorexant	67.3	38.0–119.2	12 (1.8)
Quetiapine	53.3	43.0–66.1	97 (14.3)
Zopiclone	43.6	24.6–77.2	12 (1.8)
Armodafinil	40.8	16.9–98.4	5 (0.7)
Amfetamine; Dexamfetamine	33.4	17.9–62.4	10 (1.5)
Temazepam	32.7	13.6–78.9	5 (0.7)
Trazodone	25.5	15.0–43.4	14 (2.1)
Modafinil	24.0	7.7–74.8	3 (0.4)
Promethazine	21.5	10.2–45.2	7 (1.0)
Tizanidine	21.4	8.0–57.2	4 (0.6)
Lisdexamfetamine	20.3	9.6–42.8	7 (1.0)
Ziprasidone	16.9	7.6–37.7	6 (0.9)
Eszopiclone	15.6	5.8–41.6	4 (0.6)
Aripiprazole	15.4	10.2–23.1	24 (3.6)
Buspirone	13.8	4.4–43.0	3 (0.4)
Pramipexole	13.4	5.0–35.9	4 (0.6)
Topiramate	13.4	7.2–25.0	10 (1.5)
Ethanol	11.8	5.6–24.8	7 (1.0)
Vortioxetine	11.6	4.3–30.9	4 (0.6)
Duloxetine	11.3	7.1–18.1	18 (2.7)
Escitalopram	10.3	5.4–20.0	9 (1.3)
Phenobarbital	10.3	3.3–31.9	3 (0.4)
Lurasidone	10.3	3.3–31.9	3 (0.4)
Phentermine	10.2	4.3–24.7	5 (0.7)
Clonazepam	9.9	5.1–19.2	9 (1.3)
Venlafaxine	8.8	5.2–15.0	14 (2.1)
Hydrochlorothiazide	7.4	3.1–17.8	5 (0.7)
Mirtazapine	7.0	2.9–16.9	5 (0.7)
Olanzapine	6.6	3.6–12.0	11 (1.6)
Alprazolam	5.6	2.7–11.8	7 (1.0)
Fluoxetine	5.0	2.6–9.6	9 (1.3)
Methylphenidate	4.6	1.9–11.0	5 (0.7)
Sertraline	4.3	2.2–8.7	8 (1.2)

ROR: Reporting Odds Ratio; CI: Confidence Interval.

## Data Availability

The data that support the findings of this study are available from Uppsala Monitoring Center (UMC) but restrictions apply to the availability of these data, which were used under license for the current study, and so are not publicly available. Access to VigiBase^®^ is available without fees to Dr. Fanny Rocher. Data are however available from the authors upon reasonable request and with permission of UMC.

## Supplementary Materials

The following supporting information can be downloaded at: https://www.mdpi.com/article/10.3390/jcm11133890/s1, Table S1: Reported suspected drugs in patients with SRED.
