# Supplementary material for: Medications as a Trigger of Sleep-Related Eating Disorder: A Disproportionality Analysis
Source: J Clin Med. 2022 Jul 4;11(13):3890. doi: 10.3390/jcm11133890 (PMC9267629; doi:10.3390/jcm11133890)
Supplement: Supplementary file 1 [file jcm-11-03890-s001.zip › jcm-1757839-supplementary.pdf]

Table S1. Reported suspected drugs in patients with SRED

Drugs	Number (%)
Zolpidem	243 (35.9)
Oxybate sodium	185 (27.4)
Quetiapine	97 (14.3)
Aripiprazole	24 (3.6)
Duloxetine	18 (2.7)
Venlafaxine	14 (2.1)
Trazodone	14 (2.1)
Zopiclone	12 (1.8)
Suvorexant	12 (1.8)
Olanzapine	11 (1.6)
Amfetamine;Dexamfetamine	10 (1.5)
Topiramate	10 (1.5)
Clonazepam	9 (1.3)
Fluoxetine	9 (1.3)
Escitalopram	9 (1.3)
Sertraline	8 (1.2)
Alprazolam	7 (1.0)
Promethazine	7 (1.0)
Lisdexamfetamine	7 (1.0)
Ethanol	7 (1.0)
Valproic acid	6 (0.9)
Ziprasidone	6 (0.9)
Methylphenidate	5 (0.7)
Armodafinil	5 (0.7)
Hydrochlorothiazide	5 (0.7)
Temazepam	5 (0.7)
Mirtazapine	5 (0.7)
Phentermine	5 (0.7)
Bupropion	4 (0.6)
Lamotrigine	4 (0.6)
Pramipexole	4 (0.6)
Pregabalin	4 (0.6)
Tizanidine	4 (0.6)
Risperidone	4 (0.6)
Eszopiclone	4 (0.6)
Oxycodone	4 (0.6)
Vortioxetine	4 (0.6)
Covid-19 vaccine	4 (0.6)
Modafinil	3 (0.4)
Metoprolol	3 (0.4)
Naproxen	3 (0.4)
Lisinopril	3 (0.4)
Tramadol	3 (0.4)
Lithium	3 (0.4)

Buspirone	3 (0.4)
Levocetirizine	3 (0.4)
Lurasidone	3 (0.4)
Codeine	3 (0.4)
Phenobarbital	3 (0.4)
Adalimumab	3 (0.4)
